# Taking a Weight History – Using Mnemonics to Learn a Missing Skill in Medical Education

**DOI:** 10.15694/mep.2017.000215

**Published:** 2017-11-29

**Authors:** Robert Kushner

**Affiliations:** 1 Northwestern University Feinberg School of Medicine

**Keywords:** weight, obesity, history

## Abstract

This article was migrated. The article was marked as recommended.

A major challenge facing medical educators today is to adequately train current and future physicians in the prevention and treatment of obesity, a key contributor to the global non-communicable disease burden. One of the central skills to be performed in the clinical encounter is to conduct a thorough and informative obesity-related history. Recent studies have confirmed the importance of the timing, pattern and trajectory of body weight as a predictor of multiple co-morbid conditions as well as future obesity. To provide a more structured and pedagogical approach to learning how to take a meaningful weight history, the familiar mnemonic “OPQRST” can be applied to facilitate this important element of the patient encounter.

## Introduction

Obesity continues to be a major national and global health challenge and a risk factor for an expanding set of non-communicable diseases, including cardiovascular disease, diabetes, chronic kidney disease, nonalcoholic fatty liver disease, metabolic syndrome, and many cancers, among other conditions. In response to the impact of obesity on our patients’ health and the advancements in the science of obesity, the Association of American Medical Colleges (AAMC) issued a report in 2007 calling for the incorporation of obesity education into the medical curriculum ( AAMC 2007). Yet despite the need to acquire the knowledge and communication skills necessary to assess and counsel patients with obesity, educational initiatives are infrequently included in undergraduate and graduate medical education. Among the multiple competencies required for obesity care is the ability to take a well-structured and informative weight history. This *Practical Tips and Guidelines* paper summarizes the importance of taking a weight history and introduces a convenient mnemonic that can be incorporated into clinical skills training.

## Importance of the Weight History

Multiple population studies have recently shown that the timing, pattern and trajectory of body weight gain is predictive of future occurrence of obesity, development of co-morbidities, physical impairment and mortality. The greatest risk of weight gain occurs during young adulthood and those who experience early and rapid weight gain are the most likely to be on a steeper trajectory and greater risk for obesity-related conditions ( Zheng et al. 2017). Using latent-class growth modeling, it has been possible to identify patterns of obesity that are predictive of insulin resistance, diabetes, metabolic syndrome, cardiovascular disease, and obesity-related cancers ( Vistisen et al 2014, Song et al 2015). Some studies have also shown that maximum attained body weight is associated with greater mortality rate ( Yu et al 2017) and in individuals with coronary artery disease, fluctuations in body weight is associated with increased mortality and cardiovascular events ( Bangalore 2017). Thus, assessing the body weight pattern and trajectory may be predictive of worsening obesity along with increased morbidity and mortality.

In addition to assessing these predictive factors, a body weight history is also useful to identify potential biological, behavioral, or psychosocial events that were associated with the weight gain. For many patients, weight gain occurs or is accelerated during pregnancy, smoking cessation, as an unintended consequence of a medication, or during a life event, such as a change in marital status or occupation. Changes in diet, physical activity, sleep patterns and stress that are coincident to these events can be determined by further probing. Additionally, the patient can be queried about any volitional weight loss efforts taken such as attending a commercial weight loss program, following a specified dietary regime, engaging in exercise, or taking an anti-obesity medication. Obtaining this information is important in having a deeper understanding of the etiology of weight gain for a particular patient and beginning to formulate a treatment plan. Thus far, no structured framework exists to take an organized and informative weight history for the assessment of obesity.

## Using mnemonics to learn weight history skills

As noted, the skill and parameters of taking a weight history have not been traditionally included in medical education. The mnemonic ‘OPQRST’ is commonly used for learning about a patient’s chief complaint, and the same mnemonic can be applied to taking a weight history ( Table 1). By asking targeted and purposeful questions, the following 5 features of the patient’s weight history can be obtained: onset, precipitating events, quality of health, remedy, setting, and temporal pattern. Examples of questions that can be used to explore these features are provided in the table. These features provide a contextual understanding of how and when patients gained weight, what efforts were employed to take control, and the impact of body weight on their health. Furthermore, by using a narrative ( Charon 2001) or autobiographical approach to obtaining the weight history, patients are able to express, in their own words, a life course perspective of the underlying burden, frustration, struggle, stigma or shame associated with trying to manage body weight. Listening should be unconditional and nonjudgmental. By letting patients tell their story, the clinician is also able to assess the patients’ awareness, knowledge, motivation, decision-making, and resiliency regarding weight management. The narrative provides a basis for approaching the patients’ weight holistically, as well as beginning to formulate diagnostic and therapeutic options ( Greenhalgh & Hurwitz 1999). If time permits, an alternative technique is to ask patients to draw their own lifestyle events - body weight graph that can be used as a conversation map to discuss their weight history ( Kushner & Ryan 2014). Using this approach which is displayed in the figure, patients sketch their weight curve over time and insert life events or treatments that they feel were temporally related. If the weight history is conducted properly, patients should feel validated and acknowledged regarding their weight journey, while the clinician should feel more empathetic and informed to provide meaningful and practical patient-centered treatment.

**Table 1.** Using the mnemonic ‘OPQRST’ to take the weight history

	Sample Questions
**Onset**	“When did you first begin to gain weight?” “What did you weight in high school, college, early 20s, 30s, 40s?” “What was your heaviest weight?”
**Precipitating**	“What life events led to your weight gain, e.g., college, long commute, marriage, divorce, financial loss?” “How much weight did you gain with pregnancy?” “How much weight did you gain when you stopped smoking?” “How much weight did you gain when you started insulin?”
**Quality of life**	“At what weight did you feel your best?” “What is hard to do at your current weight?”
**Remedy**	“What have you done or tried in the past to control your weight?” “What is the most successful approach you tried to lose weight?” “What do you attribute the weight loss to?” “What caused you to gain your weight back?”
**Setting**	“What was going on in your life when you last felt in control of your weight?” “What was going on when you gained your weight?” “What role has stress played in your weight gain?” “How important is social support or having a buddy to help you?”
**Temporal pattern**	“What is the pattern of your weight gain?” “Did you gradually gain your weight over time, or is it more cyclic (yo-yo)?” “Are there large swings in your weight, and if so, what is the weight change?”

## Take Home Messages

Taking a body weight history is an essential feature of the obesity assessment and should be included in training for all health professionals. Appling a mnemonic that is commonly used in history taking can facilitate learning this important new skill.

## Notes On Contributors

Dr. Robert Kushner is a Professor of Medicine at Northwestern University Feinberg School of Medicine, and Director of the Center for Lifestyle Medicine at Northwestern Medicine in Chicago, IL
