# Supplementary material for: Taking a Weight History – Using Mnemonics to Learn a Missing Skill in Medical Education
Source: MedEdPublish (2016). 2017 Nov 29;6:215. [Version 1] doi: 10.15694/mep.2017.000215 (PMC10885228; doi:10.15694/mep.2017.000215)
Supplement: Peer review history

# Reviewer report: Reviewer response for version 1

**DOI:** 10.21956/mep.19398.r28451

**Published:** 2017-12-01

**Authors:** Sandra Kemp

**Affiliations:** 1 Curtin Medical School, Curtin University

This review has been migrated. The reviewer awarded 3 stars out of 5 This paper provides a description of a teaching tool that could be used in clinical skills training to help medical students take an obesity-related history. As the author highlights, this is an important skill.Mnemonics are useful at times for learners and a mnemonic to take weight history is likely to be helpful, particularly early on in the training of medical students.The author has highlighted the importance of letting patients tell their story and it would have been valuable if there were further detail about how this works in the context of the mnemonic. The mnemonic itself raised questions in my mind from the patient perspective. Some of the questions related to the first letters of the mnemonic could be better placed later in the history taking, rather than at the beginning. It might be useful to start with what a patient is likely to feel comfortable talking about first, to inform the development of a mnemonic.

Reviewer Expertise:

NA

No decision status is available

# Reviewer report: Reviewer response for version 1

**DOI:** 10.21956/mep.19398.r28449

**Published:** 2017-12-01

**Authors:** Trevor Gibbs

**Affiliations:** 1 AMEE

This review has been migrated. The reviewer awarded 4 stars out of 5 A very interesting paper that describes a novel approach to taking a history regarding very important aspect of health. I found it very helpful and I am sure that students would find this helpful too. One worry I have with structured approaches to history taking ( although the questions are important) is how they are incorporated into a more two-way flowing exploration of patients feelings etc- I suppose this might come later as students become more comfortable and confident in their communication skills.I would however recommend this paper to those involved in obesity issues as well as to those involved in undergraduate communication skills.

Reviewer Expertise:

NA

No decision status is available

# Reviewer report: Reviewer response for version 1

**DOI:** 10.21956/mep.19398.r28450

**Published:** 2017-11-30

**Authors:** BALAJI ARUMUGAM

**Affiliations:** 1 TAGORE MEDICAL COLLEGE AND HOSPITAL

This review has been migrated. The reviewer awarded 3 stars out of 5 Thanks to the author for the nice mnemonic and it will be very useful for the day to day practice for the general practitioners and also for the students.

Reviewer Expertise:

NA

No decision status is available
